# The Engineering Side of Hospital Work

**Published:** 1905-05-27

**Authors:** 


### HOSPITAL ADMINISTRATION.

### CONSTRUCTION AND ECONOMICS.

### THE ENGINEERING SIDE OF HOSPITAL WORK.

(*Continued from page* 126.)

#### XIII. ELECTRICAL APPARATUS.

ELECTRICAL THEORIES.

THE other theory of electricity, which is due very largely to Professor J. J. Thomson of Cambridge, and with which Sir Oliver Lodge, and the leading pure scientists of this country are associated, is practically a return to the corpuscular theory that ruled, in connection with light, in Newton's days. It has been built up, as indicated in the preceding article, upon the observations of laboratory experiments, principally those in connection with the Röntgen Rays, and it is for this reason the writer is giving an explanation of its features. He believes that it may be easier to follow some of the phenomena in connection with the $x$-rays, and similar apparatus, if this theory is understood. As mentioned in the previous article, Heinrich Hertz made the important discovery that electric waves pass in the ether, much as heat and light waves do. Following this discovery, Hertz and his assistant, Lenard, made other discoveries, some of which had already been noticed by Crookes, in this country, the most important of which was the fact that minute particles of matter are shot off, with a very high velocity, from the cathode, in a vacuum tube. The cathode, as will be explained, is the pole or plate at which the electric current leaves a cell, tube, etc. These minute bodies were found to be charged with electricity, in a negative sense, and to have certain remarkable properties, that will be dealt with later on. From this, and from the behaviour of electric currents in the class of liquids known as electrolytes—those the current splits up into two groups—the electrotonic or corpuscular theory of electricity has been built up. On the accepted theories of chemical science, the molecule is the smallest quantity of any element or compound that can exist alone, and the atom is the smallest quantity of any element that can enter into combination. The atom is the quantity, some multiple of which is concerned in the formation of all chemical compounds. The molecule, as calculated by Lord Kelvin, is a very minute body indeed. An enormous number of molecules will go into a very small space, and obviously the atom is very much smaller than the molecule. But the electrotonic theory assumes the existence of bodies very much smaller than either the molecule or the atom. The atom is supposed to be divided up into an enormous number of very minute bodies, called variously, electrons, or corpuscles. The atom is assumed to be spherical in form, the negative electrons being grouped on the outer surface, and forming a shield for the positive electrons. It is the negative electrons, according to the theory, which are in evidence, that make known the presence of electricity, and to which are due the phenomena we are familiar with. When one or more electrons become detached from the atom, the latter becomes positively electrified, and exerts an attraction for any atom that is negatively electrified, and for any negative electrons that it meets. The positive electrons, according to the theory, never appear, and only exert the negative force referred to above. An electric current, on this theory consists of a procession of negative electrons passing along a conductor. The theory will be referred to again in the course of these articles, but there are certain points that may be mentioned, which apparently go to confirm the theory. A charged body in motion will create all the phenomena of magnetism, etc., that are the properties of electric currents. Also it has been observed that there is a mechanical action of electric currents, very much like the action referred to of the motion of the particles emanating from the cathode in the Crookes tube. In the electrical incandescent lamp, for instance, from the moment the lamp is put into service, a bombardment commences from the filament, of very finely divided carbon, on to the inner surface of the glass. In the arc also, there is a distinct mechanical action, by which minute quantities of the anode are carried over, sometimes to the cathode, and sometimes merely into the intervening space, but it will be observed that these are emanations from the anode, not the cathode. The writer will leave the subject of electrical theories at this point, referring to them from time to time, as occasion requires. The attitude of the practical engineer towards the latest theory is that of suspended judgment. The theory which ultimately survives must be able to explain every phenomena that is met with, and to answer every question which the advance of knowledge, and constant experiment propound. It is not always appreciated, perhaps seldom, by pure scientists, that the practical engineer must necessarily be the final judge of any theory, and that from his judgment there is no appeal. As mentioned in the previous article, the electrician who represents so-called pure science lives in another world, electrically speaking, from the practical engineer. Too often he has a somewhat unhealthy contempt for facts.

THE PROPERTIES OF ELECTRICITY. ELECTRIC CURRENTS.

The most important property, and that which the writer wishes to impress upon his readers as forcibly as he knows how, is that wherever a difference of electrical pressure exists between any two points or between any two surfaces, currents of electricity will pass between the two points, or the two surfaces by every path that is open to the difference of pressure. It is the latter portion of the above law that the writer particularly wishes to impress. In the early days of electrical work it was common to hear so-called practical men of those days say "The current will never leave copper for iron." Later it was common to hear it said that the current would never leave the copper conductor for its rubber or other insulating envelope. Both were equally wrong. If there are two or more paths for the current, one of copper and others of iron or other substances, the current will pass through the copper and through the iron and other substances. Probably the current which passes through the copper will be stronger than that which passes through the iron, but not necessarily so. Again, while the greater part of the current will pass through the copper of a cable, some will and does pass through the insulating envelope, and it is the existence of this leakage current, as it is termed, that gives the electric light and power engineer some of his most troublesome difficulties. The matter is of importance in connection with medical and surgical work, because the same ideas exist, in some minds, in connection with the passage of electric currents through the tissues. The blood-vessels are undoubtedly the best conductors in the human and in most animal bodies, but every other substance conducts to some extent, and will take its current in proportion to its ability to conduct. The bones, the walls of the great blood-vessels, and the finest capillaries will all conduct, and there will be currents passing in each of them whenever a difference of electrical pressure exists between any portions of them. All possible paths must be taken into consideration in connection with the action of electric currents on and in the body. Thus the surface of the skin forms one path, and often, as will be explained later, perhaps the most important path. A striking instance of the above is seen in the method that is adopted by his Majesty's Post Office Department, for communicating without wires between certain islands off the coast and the mainland by telephone. A battery with telephonic apparatus is fixed on the mainland and another on the island in each case, and each set of apparatus is connected to wires leading to metal plates immersed in the sea. The calling and speaking currents pass from the battery on each side to the water, and across the water to plates and the wires leading to the apparatus on the other side. If the old supposition had been true, that the current passed by the shortest path, and only by that path, there would be no current passing across the water between the two sets of plates, it would have passed only from one plate to the other on its own side. What actually happens is, currents do pass between the two plates on the same side, but currents also pass outwards to the sea in curves, reaching more and more outwards, and the plates on the other side receive current because they form part of one of these curves. The practical results of this may be understood when it is stated that speech is transmitted in this manner across some eight miles of sea-water on the Irish coast. The current that passes through the insulating envelope of the electric light and power cables though small in quantity is far from negligible, no matter how carefully the installation may be designed, and it has led, in several cases to the deterioration of the envelope and the breakdown of the cable. An electric current, however small, always does work; another point the writer wishes to impress upon the readers of these articles. The passage of an electric current implies the delivery of a certain quantity of energy, and this energy must re-appear, in some form or other, no matter how small it may be in quantity.

*(To be continued.)*

